# Association Between Socioeconomic Status and the Prevalence of Metabolic Diseases: A Nationwide Cross‐Sectional Study in China

**DOI:** 10.1111/1753-0407.70222

**Published:** 2026-04-07

**Authors:** Minghan Zhang, Junru Fan, Qiuyu Cao, Ruizhi Zheng, Yu Xu, Min Xu, Feixia Shen, Xuejiang Gu, Yiming Mu, Lulu Chen, Tianshu Zeng, Lixin Shi, Qing Su, Xuefeng Yu, Li Yan, Guijun Qin, Qin Wan, Gang Chen, Xulei Tang, Zhengnan Gao, Ruying Hu, Zuojie Luo, Yingfen Qin, Li Chen, Xinguo Hou, Yanan Huo, Qiang Li, Guixia Wang, Yinfei Zhang, Chao Liu, Youmin Wang, Shengli Wu, Tao Yang, Huacong Deng, Jiajun Zhao, Guang Ning, Jieli Lu, Weiqing Wang, Yufang Bi, Yuhong Chen, the 4C Study Group

**Affiliations:** ^1^ Department of Endocrine and Metabolic Diseases, Shanghai Institute of Endocrine and Metabolic Diseases, Ruijin Hospital Shanghai Jiao Tong University School of Medicine Shanghai China; ^2^ Shanghai National Clinical Research Center for Endocrine and Metabolic Diseases, Ruijin Hospital Shanghai Jiao‐Tong University School of Medicine Shanghai China; ^3^ The First Affiliated Hospital of Wenzhou Medical University Wenzhou China; ^4^ Chinese People's Liberation Army General Hospital Beijing China; ^5^ Union Hospital, Tongji Medical College Huazhong University of Science and Technology Wuhan China; ^6^ Affiliated Hospital of Guiyang Medical College Guiyang China; ^7^ Xinhua Hospital Affiliated to Shanghai Jiaotong University School of Medicine Shanghai China; ^8^ Tongji Hospital, Tongji Medical College Huazhong University of Science and Technology Wuhan China; ^9^ Sun Yat‐sen Memorial Hospital Sun Yat‐sen University Guangzhou China; ^10^ The First Affiliated Hospital of Zhengzhou University Zhengzhou China; ^11^ The Affiliated Hospital of Southwest Medical University Luzhou China; ^12^ Fujian Provincial Hospital Fujian Medical University Fuzhou China; ^13^ The First Hospital of Lanzhou University Lanzhou China; ^14^ Central Hospital of Dalian University of Technology Dalian China; ^15^ Zhejiang Provincial Center for Disease Control and Prevention Hangzhou China; ^16^ The First Affiliated Hospital of Guangxi Medical University Nanning China; ^17^ Qilu Hospital of Shandong University Jinan China; ^18^ Jiangxi Provincial People's Hospital Affiliated to Nanchang University Nanchang China; ^19^ The Second Affiliated Hospital of Harbin Medical University Harbin China; ^20^ The First Hospital of Jilin University Changchun China; ^21^ Central Hospital of Shanghai Jiading District Shanghai China; ^22^ Jiangsu Province Hospital on Integration of Chinese and Western Medicine Nanjing China; ^23^ The First Affiliated Hospital of Anhui Medical University Hefei China; ^24^ Karamay Municipal People's Hospital Xinjiang China; ^25^ The First Affiliated Hospital of Nanjing Medical University Nanjing China; ^26^ The First Affiliated Hospital of Chongqing Medical University Chongqing China; ^27^ Shandong Provincial Hospital Affiliated to Shandong University Jinan China

**Keywords:** metabolic diseases, prevalence, socioeconomic status

## Abstract

**Background:**

Metabolic diseases remain a fast‐growing global health burden. Besides other known factors, socioeconomic status (SES) has been recognized as a key determinant of metabolic health. This study aimed to investigate the association between SES and the prevalence of metabolic diseases in the Chinese population.

**Methods:**

We analyzed data from a nationwide community‐based cross‐sectional study in China. SES was derived to a composite score (range 0–3) using three indicators (educational attainment, living conditions, and marital status), with higher scores indicating better SES. Chronic diseases were diagnosed through biochemical testing and clinical assessments. Logistic regression models were applied to estimate associations between SES scores and metabolic diseases.

**Results:**

Among 201 532 participants, the lowest SES group had 87.1% higher odds of metabolic diseases than the highest SES group (odds ratio [OR] = 1.871, 95% confidence interval [CI]: 1.739, 2.016). One‐point decrease in SES score was associated with increased prevalence of hypertension (OR = 1.096, 95% CI: 1.080, 1.113) and obesity (OR = 1.112, 95% CI: 1.091, 1.134). In contrast, lower SES scores were linked to a reduced prevalence of dyslipidemia (OR = 0.931, 95% CI: 0.918, 0.945) and diabetes (OR = 0.974, 95% CI: 0.958, 0.990) after adjusting for covariates. We also observed that lower SES scores showed stronger associations with increased prevalence of obesity and hypertension in women but decreased risks of diabetes, dyslipidemia, and obesity in men.

**Conclusions:**

Lower SES was associated with a higher prevalence of hypertension and obesity but a lower prevalence of dyslipidemia and diabetes, with significant gender differences.

## Highlights

- This large national cross‐sectional study revealed SES differentially affects the prevalence of various metabolic diseases.
- Low SES was significantly associated with a higher prevalence of hypertension and obesity, but inversely associated with diabetes and dyslipidemia.
- Gender‐specific variations existed in the associations between SES and metabolic diseases.
- Strategies for preventing metabolic diseases should comprehensively account for SES and deliver tailored interventions.

## Graphical abstract

In a nationwide community‐based cross‐sectional study of 201,532 Chinese adults, lower socioeconomic status was associated with a greater overall burden of metabolic diseases, with distinct patterns across diabetes, dyslipidemia, hypertension, and obesity.

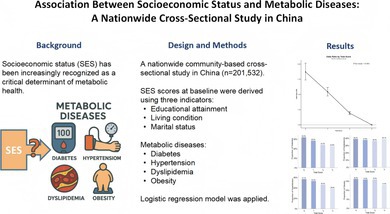

## 1 Introduction

Metabolic diseases, including hypertension, type 2 diabetes, obesity, and dyslipidemia, have gradually developed as major public health problems threatening people's health, especially in low‐ and middle‐income countries [1]. These metabolic disorders share common pathophysiological mechanisms and interrelated risk factors [2]. While existing research mainly focused on biological determinants, evidence on the associations between upstream socioeconomic factors, which may impact downstream access to material resources and behavioral outcomes, and metabolic diseases remains limited [3].

Socioeconomic status (SES) reflects the cumulative effect of external factors on human health, including living environment, personal qualities, and behavioral patterns [4]. The associations between SES and metabolic diseases vary across countries at different development stages [5]. It has been shown that in developed countries, metabolic diseases are more common among individuals with lower SES, whereas in developing countries, these diseases tend to be more prevalent in those with middle or higher SES [6]. Different dimensions of SES, educational attainment, marital status, and living conditions affect health outcomes through different mechanisms [7]. Education typically reflects an individual's level of health literacy, knowledge, and skills [8]. Those with higher levels of education tend to have better socioeconomic resources and are more likely to adopt health‐promoting behaviors and lifestyles [9, 10]. Living alone may weaken the social support network and reduce health behavior regulation, lead to elevated psychosocial stressors and financial limitations, which increases susceptibility to metabolic diseases [11, 12]. Conversely, partnered marital status typically provides both affective reinforcement and pooled socioeconomic assets, enabling reciprocal health maintenance through spousal health advocacy, consequently attenuating morbidity patterns [13]. While several studies in China have investigated the association between SES and metabolic diseases [14, 15, 16], the current evidence remains limited due to generally small sample sizes and substantial heterogeneity in findings across studies. In addition, the impact of SES on metabolic diseases could be different in men and women, which has been reported in several studies [17, 18], further research is needed to clarify these differential patterns.

Therefore, in this study, we used data from the Risk Evaluation of cAncers in Chinese diabeTic Individuals: a lONgitudinal (REACTION) study to explore the associations of SES, assessed by educational attainment, marital status, and living condition, with the prevalence of metabolic diseases. In addition, we explored whether such correlations differed in men and women.

## 2 Methods

### 2.1 Study Design and Participants

Risk Evaluation of cAncers in Chinese diabeTic Individuals: a lONgitudinal (REACTION) study was a nationwide community‐based cohort study, which has been previously described elsewhere [19]. The baseline survey was conducted from 2011 to 2012. A total of 259 657 adults were recruited from 25 communities covering 16 provinces across mainland China. For this study, we excluded participants with missing data on educational attainment, marital status, and living conditions (*n* = 7977), as well as those with incomplete covariate information (*n* = 50 148). Ultimately, 201 532 participants were included. The study was approved by the Medical Ethics Committee of Ruijin Hospital affiliated to Shanghai Jiao Tong University School of Medicine and was conducted in accordance with institutional guidelines. All study participants provided written informed consent.

### 2.2 Data Collection

Baseline assessments were conducted via face‐to‐face interviews utilizing a standardized questionnaire, complemented by anthropometric measurements and blood sample collection at local community clinics. We collected data on lifestyle habits, including smoking, drinking, and exercise habits, based on self‐reported data. Specific definitions and measurements are detailed in Table S1. Metabolic indicators were measured by experienced nurses according to the standard protocol. The personal interview was conducted by trained study personnel using a structured questionnaire. Body weight and height were measured by experienced nurses according to the standard protocol. Body mass index (BMI) was calculated as body weight in kilograms divided by body height in meters squared (kg/m^2^). All participants underwent an oral glucose tolerance test, and plasma glucose was obtained at zero and 2 h during the test. Blood specimens and first morning spot urine samples were collected and aliquoted into 0.5 mL Eppendorf tubes within 2 h of collection and shipped in dry ice to the central laboratory of the study located at Shanghai Institute of Endocrine and Metabolic Diseases, which is accredited by the College of American Pathologists. Three blood pressure measurements were obtained with participants in a seated position after 5 min of quiet rest using an automated electronic device (OMRON Model HEM‐752 FUZZY, Dalian, China). Fasting and 2‐h post load glucose (2h‐PG) levels were measured using a glucose oxidase method. Hemoglobin A1c (HbA1c) was measured using high‐performance liquid chromatography (VARIANT II System, Bio‐Rad Laboratories, CA, USA). Serum fasting total cholesterol, high‐density lipoprotein cholesterol (HDL‐C), and low‐density lipoprotein cholesterol (LDL‐C) were measured using an auto‐analyzer (ARCHITECT ci16200 analyzer, Abbott Laboratories, Illinois, USA). All biochemical tests were performed at the central clinical laboratory using validated and standardized protocols and procedures [20].

### 2.3 Socioeconomic Status

In this study, SES was assessed through a composite score based on three dichotomized dimensions: educational attainment, marital status, and living conditions. Educational attainment was evaluated based on participants' self‐reported highest level of education, which was subsequently classified into two categories: low (elementary school, no formal education, or middle school) or high (high school, technical school/college, or above). Marital status and living conditions were also documented through the questionnaire. Marital status was categorized into two groups: favorable (married or having a partner) or unfavorable (single, divorced, separated, or others). Living conditions were also classified into two groups: favorable (living with a spouse or children) or unfavorable (living alone). Each dimension was scored dichotomously, with 0 points assigned to low educational attainment, unfavorable marital status, and unfavorable living conditions, and 1 point assigned to all other categories. The scores from these three dimensions were summed to generate a composite SES score, ranging from 0 to 3 points. A higher SES score represents better SES.

### 2.4 Assessment of Metabolic Diseases

We defined a metabolic disease as having any one of the following: diabetes, hypertension, dyslipidemia, or obesity. The four diseases were diagnosed according to the following criteria: (a) diabetes was diagnosed if fasting plasma glucose (FPG) ≥ 7.0 mmol/L, or 2h‐PG ≥ 11.1 mmol/L, or HbA1c ≥ 6.5%, or a prior diagnosis by physicians, or under anti‐diabetes medication [21]; (b) hypertension was defined as systolic blood pressure (SBP) ≥ 140 mmHg or diastolic blood pressure (DBP) ≥ 90 mmHg using the mean of three readings, or under anti‐hypertensive medication [22]; (c) dyslipidemia was defined as triglyceride ≥ 2.26 mmol/L, or total cholesterol ≥ 6.22 mmol/L, or LDL‐C ≥ 4.14 mmol/L, or HDL‐C < 1.01 mmol/L, or under lipid‐lowering medication [23]; (d) obesity was defined as BMI ≥ 28 kg/m^2^ [24].

### 2.5 Statistical Analysis

Data on the basic characteristics are presented as means ± standard deviations (SD) or median (interquartile range) for continuous variables, and numbers (proportions, %) for categorical measures. Distributions of metabolic diseases in different SES scores subgroups are visualized by bar plot.

Logistic regression models were used to estimate odds ratios (ORs) and 95% confidence intervals (CIs) for the association between the SES scores and overall or individual metabolic diseases. Model 1 was the unadjusted model; model 2 adjusted for age and sex; model 3 adjusted for age, sex, smoking habit, drinking habit, and physical activity level. Next, we analyzed the association between each factor that constitutes the SES score (e.g., educational attainment, marital status, and living condition) and the prevalence of metabolic diseases by logistic regression.

Given that the strength of associations between educational attainment, marital status and living condition with chronic disease outcomes may vary, we constructed a weighted SES score using the following formula: weighted SES score = (β1*education + β2*marital status + β3*living condition) × (3/sum of the β coefficients). Logistic regression model was used to estimate the β coefficients, with overall metabolic disease as the outcome and SES components as independent variables [25]. This weighted score was subsequently used to assess its association with four specific metabolic diseases. We also assessed the associations between SES scores and metabolic diseases by sex and age subgroups. The interaction term between the SES scores group and age or sex was also added to obtain interaction *p*‐values.

Statistical analyses were performed with R (version 4.3.3; The R Foundation). Statistical significance level was a two‐tailed *p*‐value of < 0.05.

## 3 Results

The baseline characteristics of the participants categorized by the SES score are described in Table 1. A total of 201 532 participants (mean age 57.49, SD 9.66 years) were included from the REACTION study. Fifty five thousand nine hundred and ninety nine (27.79%) were men and 145 533 (72.21%) were women. Participants with higher SES scores were more likely to be men, younger, and have more alcohol and tobacco intake. In addition, key metabolic indicators such as fasting blood glucose, HbA1c, LDL‐C, SBP, DBP, and BMI were lower in individuals with higher SES scores compared with those with lower scores.

**TABLE 1:** Baseline characteristics of participants stratified by SES score group.

Characteristics	Overall population (*n* = 201 532)	SES score = 3 (*n* = 66 622)	SES score = 2 (*n* = 118 463)	SES score = 1 (*n* = 11 933)	SES score = 0 (*n* = 4514)
Age (years)	57.49 ± 9.66	55.37 ± 9.12	57.71 ± 9.36	63.68 ± 10.54	66.55 ± 9.68
Sex					
Male (%)	55 999 (27.79)	23 249 (34.90)	30 463 (25.72)	1601 (13.42)	686 (15.20)
Female (%)	145 533 (72.21)	43 373 (65.10)	88 000 (74.28)	10 332 (86.58)	3828 (84.80)
Physical exercise					
Moderate or High (%)	28 861 (14.32)	12 820 (19.24)	13 788 (11.64)	1584 (13.27)	669 (14.82)
Low (%)	172 671 (85.68)	53 802 (80.76)	104 675 (88.36)	10 349 (86.73)	3845 (85.18)
Smoking					
Yes (%)	32 590 (16.17)	12 127 (18.20)	18 781 (15.85)	1172 (9.82)	510 (11.30)
No (%)	168 942 (83.83)	54 495 (81.80)	99 682 (84.15)	10 761 (90.18)	4004 (88.70)
Drinking					
Yes (%)	51 673 (25.64)	20 979 (31.49)	27 606 (23.30)	2205 (18.48)	883 (19.56)
No (%)	149 859 (74.36)	45 643 (68.51)	90 857 (76.70)	9728 (81.52)	3631 (80.44)
Living condition					
Living alone (%)	8771 (4.35)	0	642 (0.54)	3615 (30.29)	4514 (100)
Living with a spouse or children (%)	192 761 (95.65)	66 622 (100)	117 821 (99.46)	8318 (69.71)	0
Marital status					
Married or having a partner (%)	183 218 (90.91)	66 622 (100)	115 027 (97.10)	1569 (13.15)	0
Single, divorced, separated or widowed (%)	18 314 (9.09)	0	3436 (2.90)	10 364 (86.85)	4514 (100)
Educational attainment					
High school or above (%)	72 746 (36.10)	66 622 (100)	4078 (3.44)	2046 (17.15)	0
Illiteracy, primary school, or middle school (%)	128 786 (63.90)	0	114 385 (96.56)	9887 (82.85)	4514 (100)
Chronic disease					
Diabetes (%)	48 661 (24.15)	15 175 (22.78)	28 639 (24.18)	3401 (28.50)	1446 (32.03)
Hypertension (%)	89 566 (44.44)	25 725 (38.61)	54 913 (46.35)	6352 (53.23)	2576 (57.07)
Obesity (%)	30 253 (15.01)	8681 (13.03)	18 935 (15.98)	1902 (15.94)	735 (16.28)
Dyslipidemia (%)	75 538 (37.48)	26 051 (39.10)	43 212 (36.48)	4506 (37.76)	1769 (39.19)
Metabolic indicators					
Fasting blood glucose (mmol/L)	5.93 ± 1.62	5.86 ± 1.51	5.95 ± 1.66	6.01 ± 1.70	6.11 ± 1.78
2‐h post load glucose (mmol/L)	8.32 ± 3.80	8.15 ± 3.64	8.34 ± 3.85	8.73 ± 3.96	9.16 ± 4.20
Hemoglobin A1c (%)	5.80 (5.50, 6.20)	5.80 (5.50, 6.20)	5.80 (5.50, 6.20)	5.90 (5.60, 6.30)	6.00 (5.70, 6.40)
Low‐density lipoprotein cholesterol (mmol/L)	2.86 ± 0.87	2.88 ± 0.87	2.85 ± 0.86	2.91 ± 0.88	2.92 ± 0.91
Triglyceride (mmol/L)	1.30 (0.93, 1.88)	1.29 (0.92, 1.88)	1.30 (0.93, 1.88)	1.33 (0.96, 1.89)	1.34 (0.97, 1.91)
Cholesterol (mmol/L)	4.93 ± 1.12	4.91 ± 1.13	4.93 ± 1.12	5.03 ± 1.16	5.05 ± 1.16
High‐density lipoprotein cholesterol (mmol/L)	1.33 ± 0.35	1.31 ± 0.35	1.34 ± 0.35	1.35 ± 0.36	1.34 ± 0.35
Systolic blood pressure (mmHg)	132.87 ± 20.82	129.09 ± 19.27	134.37 ± 21.14	136.76 ± 22.16	138.96 ± 22.31
Diastolic blood pressure (mmHg)	77.82 ± 11.07	77.17 ± 10.84	78.32 ± 11.14	76.88 ± 11.24	76.73 ± 11.10
Body mass index (kg/m^2^)	24.62 ± 3.60	24.44 ± 3.50	24.73 ± 3.63	24.58 ± 3.79	24.63 ± 3.69

Abbreviation: SES, socioeconomic status.

**FIGURE 1:**
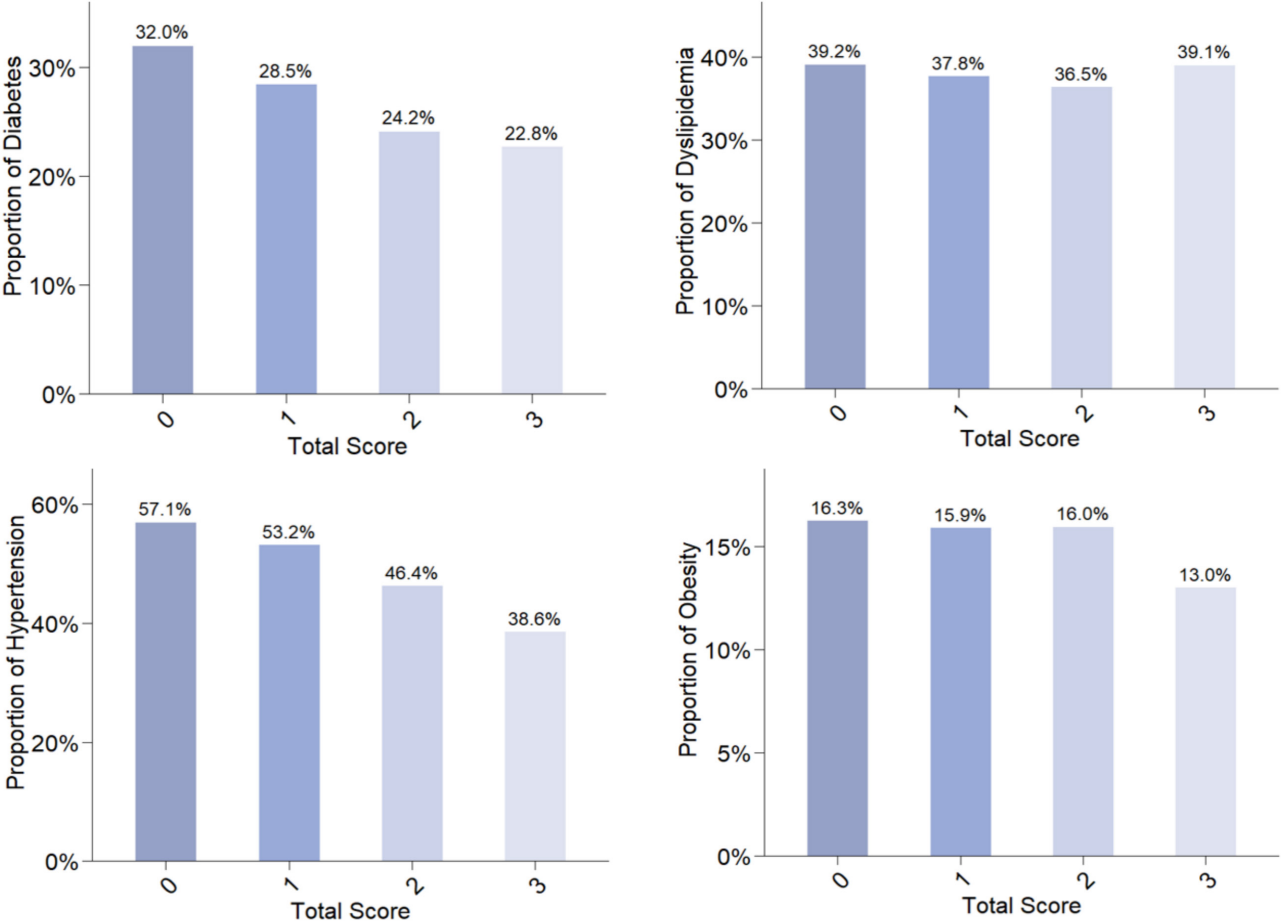
Prevalence of diabetes, dyslipidemia, hypertension, and obesity by different SES scores groups. SES, socioeconomic status.

As shown in Figure 1, the prevalence of diabetes, hypertension, and obesity decreased significantly with increasing SES scores. The prevalence of diabetes declined from 32.0% of the lower SES status to 22.8% of the higher SES, hypertension from 57.1% to 38.6%, and obesity from 16.3% to 13.0%. The prevalence of dyslipidemia was similar in different SES groups.

**FIGURE 2:**
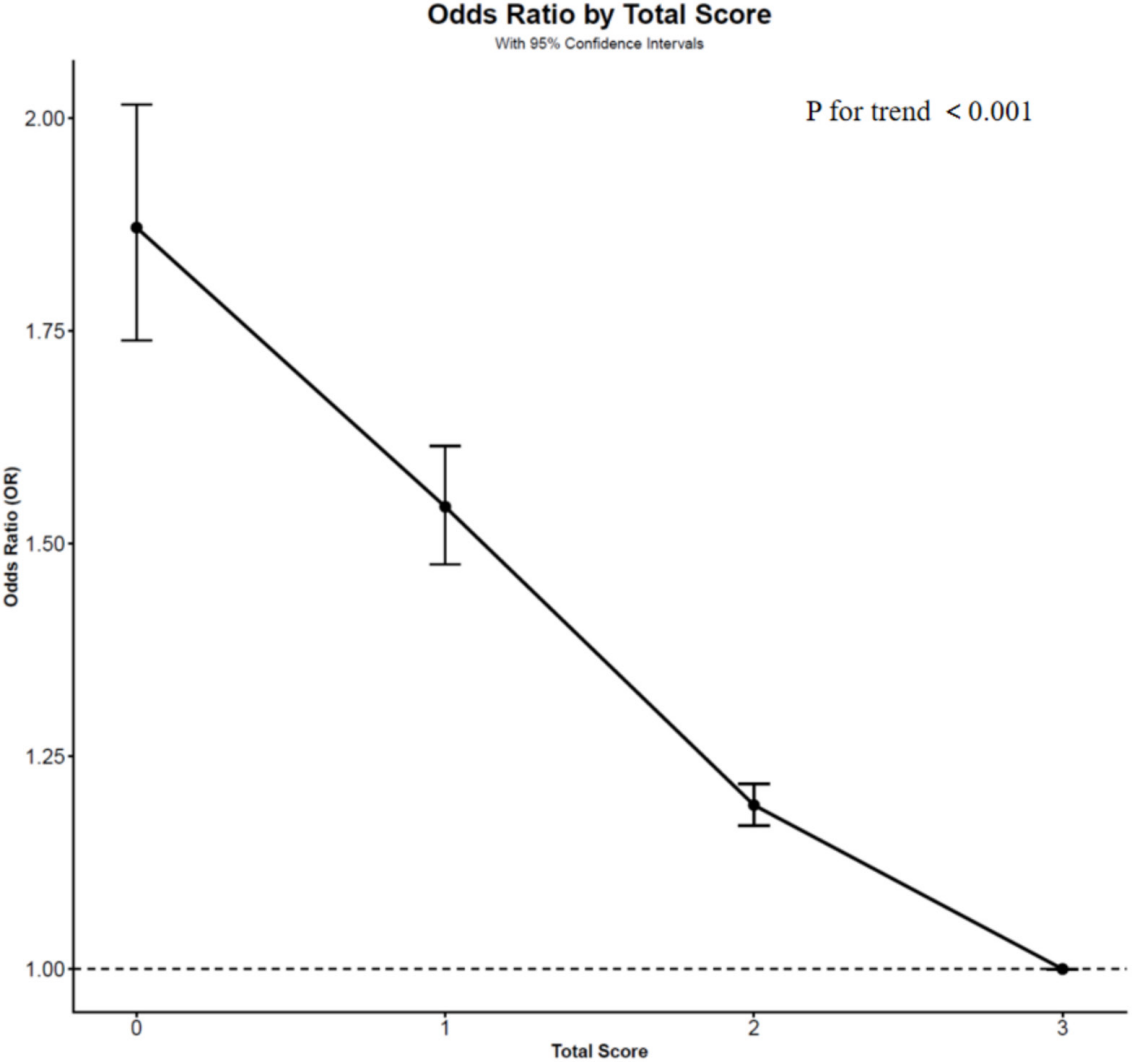
SES scores in relation to risk of metabolic diseases. CI, confidence interval; OR, odds ratio; SES, socioeconomic status. We defined participants with diabetes, hypertension, dyslipidemia, and obesity as having a chronic condition and performed a logistic regression with SES score, with ORs in the vertical and SES score in the horizontal.

Figure 2 presents the significant associations between SES scores and the prevalence of metabolic diseases. Individuals in the lowest SES group had 87.1% higher odds of metabolic diseases compared with those in the highest SES group (OR = 1.871, 95% CI: 1.739, 2.016). And the ORs (95% CIs) of metabolic diseases in people who had SES scores of 1 or 2 were 1.543 (1.475, 1.614) and 1.193 (1.168, 1.217) compared with those who had a SES score of 3.

Table 2 presents the ORs of SES scores associated with the prevalence of diabetes, dyslipidemia, hypertension and obesity after adjustment of potential confounders. In the fully adjusted model (Model 3), the associations showed a distinct pattern for different diseases. Firstly, after adjusting for age, sex and lifestyle factors, a lower SES score was associated with a decreased risk of diabetes. Each one‐point decrease in SES score was associated with a 2.6% reduction in diabetes prevalence (OR = 0.974, 95% CI: 0.958, 0.990). Secondly, a reduced SES score was linked to a decreased prevalence of dyslipidemia. The OR for each one‐point decrease in SES score was 0.931 (95% CI: 0.918, 0.945). Thirdly, each unit reduction in SES score was associated with 9.6% higher odds of hypertension (OR = 1.096, 95% CI: 1.080, 1.113). As for obesity, each unit decline in SES score was linked to an OR of 1.112 (95% CI: 1.091, 1.134) for obesity.

**TABLE 2:** Association of SES scores with prevalence of metabolic diseases.

Outcome	SES scores	Model 1 OR (95% CI)	Model 2 OR (95% CI)	Model 3 OR (95% CI)
Diabetes	SES score = 3	1 (reference)	1 (reference)	1 (reference)
	SES score = 2	1.081 (1.057, 1.106)	0.978 (0.956, 1.001)	0.974 (0.951, 0.997)
	SES score = 1	1.351 (1.294, 1.412)	0.925 (0.883, 0.969)	0.923 (0.881, 0.967)
	SES score = 0	1.598 (1.497, 1.705)	0.962 (0.898, 1.029)	0.959 (0.896, 1.027)
	Decrease per point	1.143 (1.125, 1.161)	0.976 (0.960, 0.992)	0.974 (0.958, 0.990)
Dyslipidemia	SES score = 3	1 (reference)	1 (reference)	1 (reference)
	SES score = 2	0.894 (0.877, 0.912)	0.884 (0.867, 0.902)	0.885 (0.868, 0.903)
	SES score = 1	0.945 (0.908, 0.984)	0.887 (0.851, 0.925)	0.886 (0.850, 0.924)
	SES score = 0	1.004 (0.943, 1.067)	0.904 (0.849, 0.963)	0.901 (0.846, 0.960)
	Decrease per point	0.957 (0.944, 0.970)	0.931 (0.918, 0.945)	0.931 (0.918, 0.945)
Hypertension	SES score = 3	1 (reference)	1 (reference)	1 (reference)
	SES score = 2	1.374 (1.347, 1.401)	1.227 (1.202, 1.253)	1.216 (1.191, 1.241)
	SES score = 1	1.809 (1.740, 1.882)	1.133 (1.086, 1.183)	1.127 (1.080, 1.176)
	SES score = 0	2.113 (1.988, 2.246)	1.090 (1.022, 1.164)	1.088 (1.019, 1.161)
	Decrease per point	1.332 (1.314, 1.350)	1.101 (1.085, 1.118)	1.096 (1.080, 1.113)
Obesity	SES score = 3	1 (reference)	1 (reference)	1 (reference)
	SES score = 2	1.270 (1.236, 1.305)	1.251 (1.216, 1.286)	1.230 (1.196, 1.265)
	SES score = 1	1.266 (1.199, 1.335)	1.196 (1.131, 1.264)	1.179 (1.115, 1.247)
	SES score = 0	1.298 (1.195, 1.408)	1.203 (1.105, 1.307)	1.189 (1.092, 1.292)
	Decrease per point	1.147 (1126, 1.168)	1.122 (1.101, 1.144)	1.112 (1.091, 1.134)

*Note:* Model 1 was the unadjusted model; Model 2 adjusted for age and sex; Model 3 adjusted for age, sex, smoking habit, drinking habit, and physical activity level.

Abbreviations: CI, confidence interval; OR, odds ratio; SES, socioeconomic status.

In Table S2, we conducted a sensitivity analysis to assess the associations between the weighted SES score and the four metabolic diseases. The results were consistent with the main analysis (Table 2). Moreover, we conducted sex‐stratified and age‐stratified analyses, revealing distinct gender‐specific associations (Table S3). Among men, reduced SES score correlated with a decreased prevalence of diabetes, dyslipidemia, and obesity, along with an elevated prevalence of hypertension. In contrast, among women, lower SES was associated with a lower prevalence of dyslipidemia, a higher prevalence of obesity and hypertension, and showed no significant association with diabetes. The interaction term of sex and SES score was significant (*p*‐value < 0.001). As presented in Figures S1 and S2, we found that among females, the prevalence of metabolic diseases decreased with higher SES scores (*p* for trend < 0.001). The bar charts demonstrated that females showed a decreasing prevalence of all four metabolic diseases with higher SES scores. In contrast, among males, the prevalence of hypertension followed a similar trend, whereas the prevalence of dyslipidemia progressively increased with higher SES scores. As shown in Table S4, among adults aged 40–59 years, a lower SES was associated with a higher risk of diabetes, hypertension, and obesity, but a decreased risk of dyslipidemia. Among those aged ≥ 60 years, a lower SES was associated with increased risks of hypertension and obesity but decreased risks of diabetes and dyslipidemia. There were significant differences in the associations between SES and diabetes by age.

In Table 3, we conducted a comprehensive analysis of the associations between individual components of SES and the prevalence of metabolic diseases. Lower educational attainment was linked to a 3.2% lower prevalence of diabetes (OR = 0.968, 95% CI: 0.946, 0.990) and an 11.4% lower prevalence of dyslipidemia (OR = 0.886, 95% CI: 0.869, 0.903). Conversely, it was associated with a 23.2% higher prevalence of hypertension (OR = 1.232, 95% CI: 1.208, 1.257) and a 26.3% higher prevalence of obesity (OR = 1.263, 95% CI: 1.229, 1.297). Living alone was associated with a reduced risk of metabolic diseases, with odds ratios of 0.909 (95% CI: 0.868, 0.952) in hypertension and 0.927 (95% CI: 0.872, 0.985) in obesity. However, no significant associations were observed for diabetes (OR = 1.000, 95% CI: 0.952, 1.050) or dyslipidemia (OR = 0.974, 95% CI: 0.932, 1.019). Individuals who were single, divorced, separated, or in other non‐married categories showed lower prevalence of metabolic diseases, with ORs of 0.958 (95% CI: 0.924, 0.993) for diabetes, 0.927 (95% CI: 0.897, 0.959) for hypertension, and 0.954 (95% CI: 0.913, 0.997) for obesity. No significant association was found between marital status and dyslipidemia. As shown in Table S5, gender‐specific differences were evident in the associations with the SES components. Generally, women with lower educational attainment were more likely to have increased risks of diabetes and hypertension compared to men.

**TABLE 3:** Associations of marital status, living condition, and educational attainment with four chronic diseases.

Outcome	SES factors	Model 1 OR (95% CI)	Model 2 OR (95% CI)	Model 3 OR (95% CI)
Diabetes	Educational attainment			
	High school or above	1 (reference)	1 (reference)	1 (reference)
	Illiteracy, primary school, or middle school	1.109 (1.085, 1.133)	0.972 (0.951, 0.994)	0.968 (0.946, 0.990)
	Living condition			
	Living with a spouse or children	1 (reference)	1 (reference)	1 (reference)
	Living alone	1.363 (1.300, 1.428)	0.999 (0.951, 1.049)	1.000 (0.952, 1.050)
	Marital status			
	Married or having a partner	1 (reference)	1 (reference)	1 (reference)
	Single, divorced, separated or others	1.269 (1.227, 1.313)	0.958 (0.924, 0.992)	0.958 (0.924, 0.993)
Dyslipidemia	Educational attainment			
	High school or above	1 (reference)	1 (reference)	1 (reference)
	Illiteracy, primary school, or middle school	0.903 (0.886, 0.920)	0.884 (0.868, 0.902)	0.886 (0.869, 0.903)
	Living condition			
	Living with a spouse or children	1 (reference)	1 (reference)	1 (reference)
	Living alone	1.038 (0.993, 1.085)	0.979 (0.936, 1.023)	0.974 (0.932, 1.019)
	Marital status			
	Married or having a partner	1 (reference)	1 (reference)	1 (reference)
	Single, divorced, separated or others	1.040 (1.008, 1.073)	1.001 (0.970, 1.034)	0.998 (0.966, 1.030)
Hypertension	Educational attainment			
	High school or above	1 (reference)	1 (reference)	1 (reference)
	Illiteracy, primary school, or middle school	1.444 (1.418, 1.471)	1.243 (1.219, 1.268)	1.232 (1.208, 1.257)
	Living condition			
	Living with a spouse or children	1 (reference)	1 (reference)	1 (reference)
	Living alone	1.383 (1.325, 1.443)	0.904 (0.863, 0.947)	0.909 (0.868, 0.952)
	Marital status			
	Married or having a partner	1 (reference)	1 (reference)	1 (reference)
	Single, divorced, separated or others	1.341 (1.300, 1.382)	0.924 (0.894, 0.955)	0.927 (0.897, 0.959)
Obesity	Educational attainment			
	High school or above	1 (reference)	1 (reference)	1 (reference)
	Illiteracy, primary school, or middle school	1.307 (1.274, 1.342)	1.284 (1.250, 1.318)	1.263 (1.229, 1.297)
	Living condition			
	Living with a spouse or children	1 (reference)	1 (reference)	1 (reference)
	Living alone	0.983 (0.925, 1.043)	0.924 (0.869, 0.982)	0.927 (0.872, 0.985)
	Marital status			
	Married or having a partner	1 (reference)	1 (reference)	1 (reference)
	Single, divorced, separated or others	1.010 (0.968, 1.053)	0.953 (0.912, 0.995)	0.954 (0.913, 0.997)

*Note:* Model 1 was the unadjusted model; Model 2 adjusted for age and sex; Model 3 adjusted for age, sex, smoking habit, drinking habit, and physical activity level.

Abbreviations: CI, confidence interval; OR, odds ratio; SES, socioeconomic status.

## 4 Discussion

Based on our nationwide cross‐sectional study including 201 532 middle‐aged and older Chinese adults, we comprehensively examined the relationship between SES assessed by educational attainment, marital status, and living condition and the prevalence of metabolic diseases, including diabetes, hypertension, dyslipidemia, and obesity. Individuals in the lowest SES group had 87.1% higher odds of metabolic diseases compared with those in the highest SES group. A lower SES score was associated with lower prevalence of diabetes and dyslipidemia, but associated with higher prevalence of hypertension and obesity after adjusting for age, sex, and lifestyle factors. The associations of lower SES scores with increased risks of obesity and hypertension were stronger in women, while the associations of lower SES scores with reduced risks of diabetes, dyslipidemia, and obesity were more significant in men. Additionally, educational attainment, living conditions, and marital status were associated with varying risks of metabolic diseases.

The present findings are consistent with and extend the evidence from the multinational cohort study conducted by Kivimäki et al. [26], which robustly demonstrated that disadvantaged SES significantly predicted increased risks of 18 distinct health conditions. Previous studies have consistently highlighted the increased burden of hypertension and diabetes among low‐SES populations in developing regions. For instance, a study from India by Geldsetzer et al. reported a higher tendency for hypertension and diabetes in individuals with low SES [27]. Similarly, Sung et al. documented a global shift in the prevalence of overweight/obesity toward lower‐SES groups as countries undergo economic development [28]. Additionally, data from 57 low‐ and middle‐income countries (LMICs) indicated that lipid‐related risk factors, such as elevated total cholesterol (TC), tend to worsen with lower SES [29]. However, our results present some notable divergences. After adjusting for age, sex, and lifestyle factors, we observed a higher prevalence of hypertension and obesity in low‐SES individuals, which was consistent with prior research. We found a lower prevalence of diabetes and dyslipidemia in this group, which contrasts with existing literature. These discrepancies may stem from regional variations in dietary patterns, healthcare access, or genetic predispositions [30]. For example, in our study population, lower‐SES groups might consume fewer processed foods high in refined sugars (potentially reducing diabetes risk) but still face higher obesity rates due to energy‐dense, low‐nutrient diets [31]. Similarly, the inverse association between SES and dyslipidemia could reflect differences in physical activity levels or lipid metabolism influenced by unmeasured environmental or biological factors [32].

Previous studies have shown that, compared to men, improvements in SES among women were associated with reduced prevalence of hypertension [33]. Women with moderate and high SES had a 9% (prevalence ratio: 0.91, 95% CI: 0.83, 0.98) and 30% (prevalence ratio: 0.70, 95% CI: 0.63, 0.78) lower likelihood of hypertension, respectively, compared to those with low SES, while no significant reduction was observed in men. Our study aligns with these findings, as we also observed a stronger association between lower SES and increased hypertension risk in women. Additionally, a review of 144 studies found a strong inverse relationship between SES and obesity among women in developed countries, while findings for men were inconsistent [34]. Research on the SES‐obesity relationship in developing countries remains insufficient. Our study supports this pattern, showing a stronger association between lower SES and obesity in women, filling the gap of insufficient evidence in developing countries. Regarding lipid profiles, previous studies reported that hypertriglyceridemia and low HDL cholesterol were more prevalent in men and high‐SES populations, whereas low‐SES women had a significantly higher prevalence of dyslipidemia [35]. However, our findings diverge from this by showing that lower SES was associated with a reduced risk of dyslipidemia in females. The exact mechanisms underlying these sex differences remain unclear but may be influenced by hormonal, genetic, and lifestyle factors. Similarly, NHANES data have shown that low‐SES women have a higher prevalence of diabetes and a lower prevalence in men [36]. The results of our study are consistent with it. One possible explanation is that men in lower SES groups may have greater occupational physical activity, which could help regulate glucose metabolism and reduce diabetes risk [37]. Overall, while our study confirms previous findings on the stronger associations between lower SES and obesity and hypertension in women, it also reveals novel sex‐specific differences in the relationships of SES with diabetes and dyslipidemia. These findings underscore the complex interplay between socioeconomic factors, sex, and metabolic health.

Educational attainment, marital status, and living conditions each play a distinct role in influencing the risk of metabolic diseases. It is widely recognized that lower educational attainment is associated with a higher prevalence of metabolic diseases [38]. For example, a study in 2018 highlighted that individuals with less educational attainment are more likely to develop diabetes and obesity [39]. Similarly, lower educational attainment is linked to higher rates of hypertension and dyslipidemia, as individuals with lower educational attainment are less likely to engage in regular physical activity or maintain a healthy diet [38]. However, in our study, lower educational attainment was only found to be associated with a higher prevalence of hypertension and obesity, while it was protective against diabetes and dyslipidemia. This may be due to the fact that people with low educational attainment would be less likely to undergo routine medical check‐ups for the lack of access to healthcare resources, resulting in under‐diagnosis of diabetes and dyslipidemia [40]. Marital status also significantly influences the risk of metabolic diseases. Married individuals may face a higher risk of obesity, possibly due to shared lifestyle habits [41], which is consistent with our findings. In contrast, unmarried, divorced, or widowed individuals are more likely to develop diabetes and hypertension, potentially due to social isolation and stress [42]. Different studies found varying effects of marital status on metabolic diseases, indicating the complex relationship [43, 44]. Living alone is another critical factor that influences the risk of developing metabolic diseases. Individuals who live alone often face greater social isolation and psychological distress. Several studies have shown that living alone can increase the likelihood of developing type 2 diabetes and hypertension [45, 46]. Whereas our findings found a protective effect of living alone against most metabolic diseases, a possible explanation is that people living alone tend to have greater autonomy over their health behaviors and thus are more likely to maintain healthy habits. Therefore, the association between SES and the prevalence of metabolic diseases is complex and needs to be further explored in larger prospective studies.

Our study has several strengths. Firstly, this study was a national cross‐sectional survey conducted in China with a large sample size. Secondly, we comprehensively examined the prevalence of metabolic diseases, including diabetes, hypertension, dyslipidemia, and obesity. Thirdly, the study conducted stratified analyses by age and gender, revealing distinct patterns in the relationship between SES and metabolic diseases across sexes. This detailed exploration of subgroup differences adds depth to the findings and highlights the importance of tailored interventions for specific populations. Nevertheless, this study has several limitations that should be taken into account. Firstly, the cross‐sectional design of this study limits the ability to establish a causal relationship between SES and metabolic diseases. Thus, further prospective studies are warranted to better understand the temporal relationships and potential causality. Secondly, given the unavailability of systematically collected data on household or individual income and the absence of structural economic indicators such as social security coverage, the construction of SES in this study was limited to three available and representative dimensions: educational attainment, marital status, and living conditions. Thirdly, the SES score was constructed using only three dimensions (educational attainment, marital status, and living conditions), which may not fully capture the complexity of socioeconomic status. Key indicators such as income and occupation were not included, potentially leading to an incomplete assessment of SES and its impact on metabolic diseases. Moreover, the study relied heavily on self‐reported data for socioeconomic and lifestyle variables, which may introduce recall bias and social desirability bias, potentially affecting the accuracy of the results. Lastly, although we have adjusted for several covariates, there may still be unmeasured or residual confounding factors that could influence the observed associations between SES and metabolic diseases. For example, dietary habits, stress levels, and access to healthcare were not accounted for in the analysis, which could affect the prevalence of metabolic diseases.

## 5 Conclusion

In conclusion, evidence from this large‐scale cross‐sectional study highlights the association between SES and metabolic diseases, with notable sex‐specific differences. Individuals in the lowest SES group had 87.1% higher odds of metabolic diseases compared with those in the highest SES group. A lower SES score was associated with lower prevalence of diabetes and dyslipidemia, but higher prevalence of hypertension and obesity, after adjusting for age, sex, and lifestyle factors. The associations of lower SES scores with increased risks of obesity and hypertension were stronger in women, while the associations of lower SES scores with reduced risks of diabetes, dyslipidemia, and obesity were more significant in men. Additionally, educational attainment, living conditions, and marital status were associated with varying prevalences of metabolic diseases. These findings emphasize that it is crucial to narrow the socioeconomic inequalities in metabolic diseases in China, and individualized interventions are warranted in men and women.

## Author Contributions

J.L., Y.B., Y.C., and W.W. conceived and designed the study. M.Z. and J.F. did the statistical analysis. M.Z., J.F. and Q.C. drafted the manuscript and contributed to acquisition, analysis, or interpretation of data. All authors revised the report and approved the final version before submission. J.L., Y.B., Y.C., and W.W. are the guarantors of this work and, as such, had full access to all the data in the study and take responsibility for the integrity of the data and the accuracy of the data analysis.

## Funding

This work was supported by the Noncommunicable Chronic Diseases‐National Science and Technology Major Project (2023ZD0508902, 2023ZD0508402, 2023ZD0508401, 2023ZD0508901), the Ministry of Science and Technology of the People's Republic of China (2023YFC2506704), the National Natural Science Foundation of China (82370810, 91857205, 82088102, 82372347, 82200998), the Science and Technology Committee of Shanghai (22Y31900300, 23Y11908400, 23JS1400900), the Clinical Research Project of Shanghai Municipal Health Commission (20224Y0087, 20214Y0002), and the Innovative Research Team of High‐level Local University in Shanghai.

## Disclosure

Yufang Bi, Guang Ning, and Weiqing Wang are Editorial Board members of the Journal of Diabetes and co‐authors of this article. To minimize bias, they were excluded from all editorial decision‐making related to the acceptance of this article for publication.

## Conflicts of Interest

The authors declare no conflicts of interest.

## Supporting information


**Figure S1:** Odds ratios of SES factors in relation to risk of metabolic diseases by gender.
**Figure S2:** Prevalence of diabetes, dyslipidemia, hypertension and obesity by different SES scores groups and gender.

**Table S1:** Definitions, measurement methods, and categorizations for the SES components and lifestyles.
**Table S2:** Association of weighted SES scores with prevalence of metabolic diseases.
**Table S3:** Correlation between gender‐specific SES scores and prevalence of metabolic diseases.
**Table S4:** Correlation between age‐specific SES scores and risk of four metabolic diseases.
**Table S5:** Associations of gender‐specific marital status, living condition, and educational attainment with four metabolic diseases.

## Data Availability

The data that support the findings of this study are available from the corresponding author upon reasonable request.
