# Supplementary material for: Association Between Socioeconomic Status and the Prevalence of Metabolic Diseases: A Nationwide Cross‐Sectional Study in China
Source: J Diabetes. 2026 Apr 7;18(4):e70222. doi: 10.1111/1753-0407.70222 (PMC13054516; doi:10.1111/1753-0407.70222)
Supplement: Supplementary file 1 — Figure S1: Odds ratios of SES factors in relation to risk of metabolic diseases by gender. Figure S2: Prevalence of diabetes, dyslipidemia, hypertension and obesity by different SES scores groups and gender. [file JDB-18-e70222-s002.docx]

**Figure S1. Odds ratios of SES factors in relation to risk of metabolic diseases by gender**


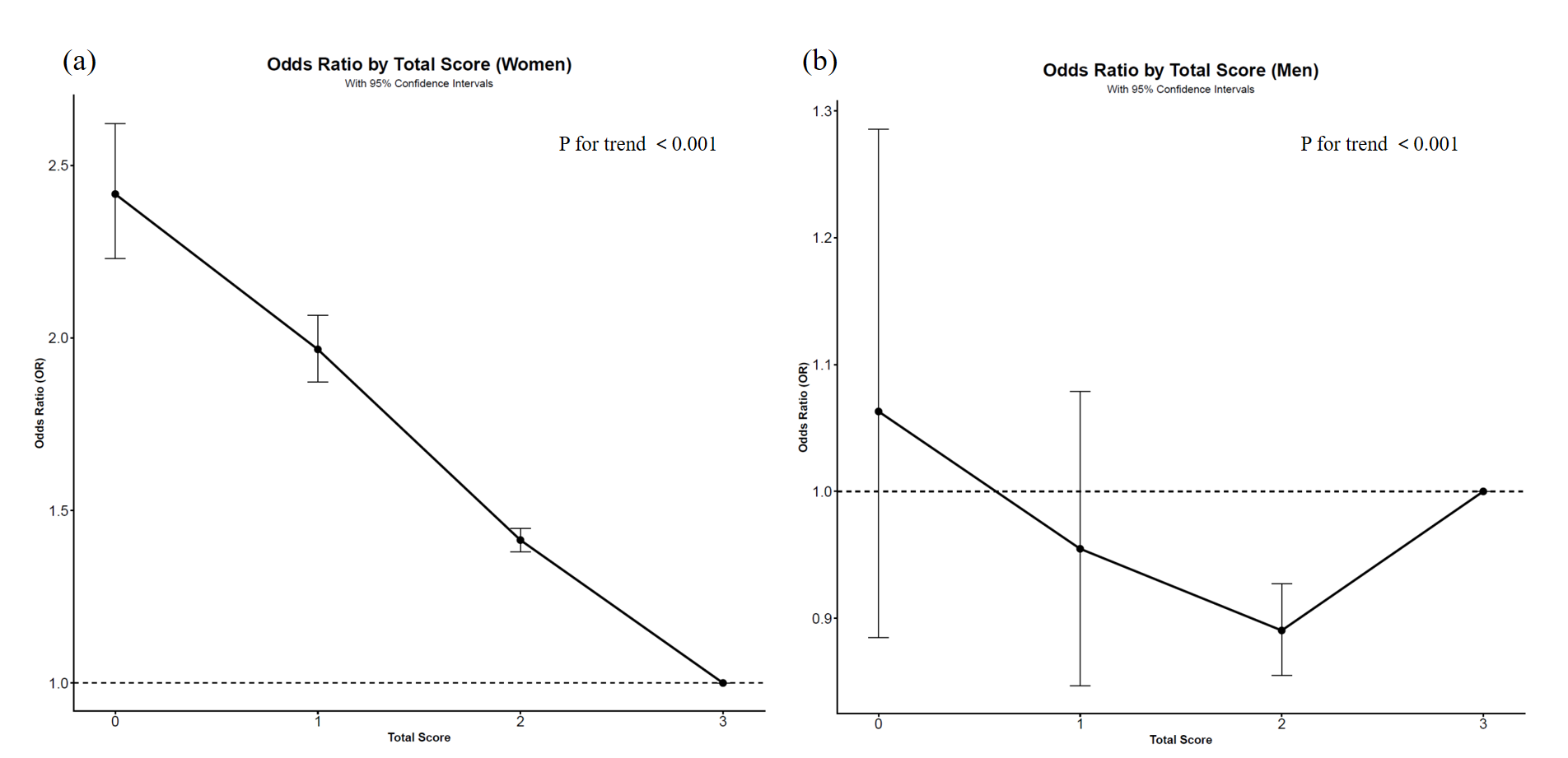


Abbreviations: SES, socioeconomic status.

**Figure S2. Prevalence of diabetes, dyslipidemia, hypertension and obesity by different SES scores groups and gender**


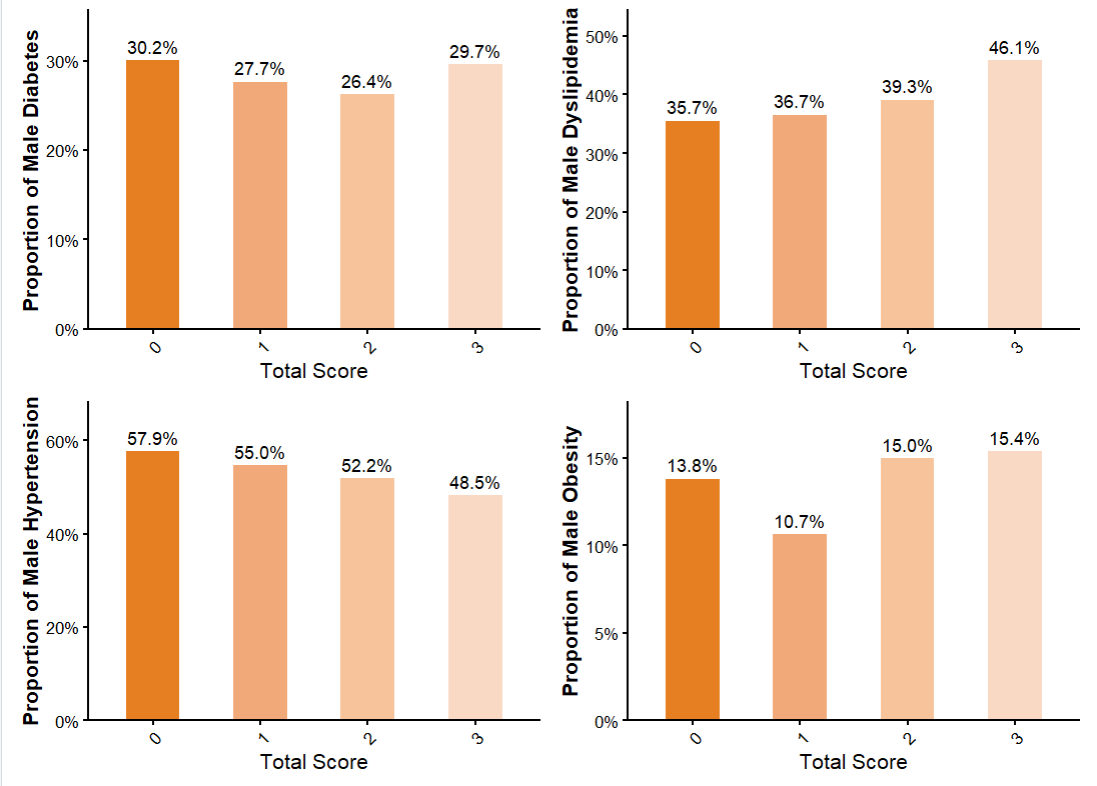

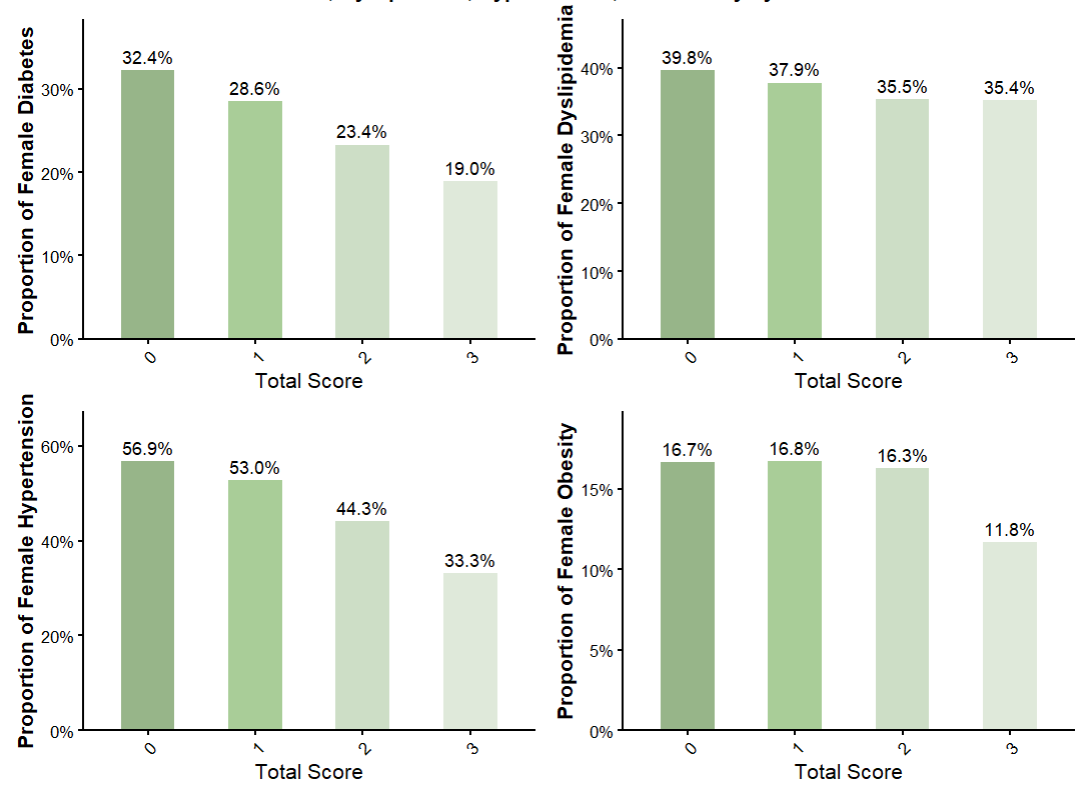


Abbreviations: SES, socioeconomic status.
