# Supplementary material for: Association Between Socioeconomic Status and the Prevalence of Metabolic Diseases: A Nationwide Cross‐Sectional Study in China
Source: J Diabetes. 2026 Apr 7;18(4):e70222. doi: 10.1111/1753-0407.70222 (PMC13054516; doi:10.1111/1753-0407.70222)
Supplement: Supplementary file 2 — Table S1: Definitions, measurement methods, and categorizations for the SES components and lifestyles. Table S2: Association of weighted SES scores with prevalence of metabolic diseases. Table S3: Correlation between gender‐specific SES scores and prevalence of metabolic diseases. Table S4: Correlation between age‐specific SES scores and risk of four metabolic diseases. Table S5: Associations of gender‐specific marital status, living condition, and educational attainment with four metabolic diseases. [file JDB-18-e70222-s001.docx]

**Table S1. Definitions, measurement methods, and categorizations for the SES components and lifestyles**

| **Category** | **Measurement and definition** | **Risk category** | **Reference category** |
| --- | --- | --- | --- |
| **Tobacco intake** | Self-reported tobacco use using a standard tobacco use frequency questionnaire.  Currently smoking daily or almost daily, or having a past smoking habit (including occasional or daily smoking).  Never tobacco use never smoked (currently not smoking and never smoked in the past). | **Current or former tobacco intake** | **Never tobacco intake** |
| **Alcohol intake** | Self-reported alcohol consumption using a standard alcohol consumption frequency questionnaire.  Currently drinking weekly or almost weekly, or having a past drinking habit (including occasional or weekly drinking).  Never alcohol intake (currently not drinking and never drank in the past). | **Current or former alcohol intake** | **Never alcohol intake** |
| **Physical activity** | Physical activity was evaluated using the International Physical Activity Questionnaire, and categorized as low (< 600 metabolic equivalents [MET] × minutes per week or < 150 minutes per week of moderate intensity physical activity), moderate (600-3,000 MET × minutes or 150-750 minutes per week) or high (> 3,000 MET × minutes or > 750 minutes per week). | Low physical activity | Moderate or high physical activity |
| **Educational attainment** | Education was self-reported, and classified into three groups: primary school or less, middle school education, or high school or above). | Low educational attainment (primary school or less, middle school education) | High school or above |
| **Marital status** | Marital status was self-reported and categorized as married or having a partner; single, divorced, separated, or widowed. | Single, divorced, separated, or widowed | Married or having a partner |
| **Living condition** | Living arrangements were self-reported and categorized as living with children and/or a spouse; or living alone. | Living alone | Living with children and/or a spouse |

Abbreviations: SES, socioeconomic status

**Table S2. Association of weighted SES scores with prevalence of metabolic diseases**

| **Outcome** | **SES scores** | **Model 1 OR (95% CI)** | **Model 2 OR (95% CI)** | **Model 3 OR (95% CI)** |
| --- | --- | --- | --- | --- |
| Diabetes | High SES score | 1 (reference) | 1 (reference) | 1 (reference) |
|  | Medium-high SES score | 1.081 (1.057, 1.106) | 0.978 (0.956, 1.001) | 0.974 (0.951, 0.997) |
|  | Medium SES score | 1.351 (1.294, 1.412) | 0.925 (0.883, 0.969) | 0.923 (0.881, 0.967) |
|  | Low SES score | 1.598 (1.497, 1.705) | 0.962 (0.898, 1.029) | 0.959 (0.896, 1.027) |
|  | Trend test | 1.129 (1.112, 1.145) | 0.977 (0.962, 0.992) | 0.975 (0.960, 0.990) |
| Dyslipidemia | High SES score | 1 (reference) | 1 (reference) | 1 (reference) |
|  | Medium-high SES score | 0.894 (0.877, 0.912) | 0.884 (0.867, 0.902) | 0.885 (0.868, 0.903) |
|  | Medium SES score | 0.945 (0.908, 0.984) | 0.887 (0.851, 0.925) | 0.886 (0.850, 0.924) |
|  | Low SES score | 1.004 (0.943, 1.067) | 0.904 (0.849, 0.963) | 0.901 (0.846, 0.960) |
|  | Trend test | 0.954 (0.941, 0.966) | 0.930 (0.918, 0.943) | 0.931 (0.918, 0.943) |
| Hypertension | High SES score | 1 (reference) | 1 (reference) | 1 (reference) |
|  | Medium-high SES score | 1.374 (1.347, 1.401) | 1.227 (1.202, 1.253) | 1.216 (1.191, 1.241) |
|  | Medium SES score | 1.809 (1.740, 1.882) | 1.133 (1.086, 1.183) | 1.127 (1.080, 1.176) |
|  | Low SES score | 2.113 (1.988, 2.246) | 1.090 (1.022, 1.164) | 1.088 (1.019, 1.161) |
|  | Trend test | 1.317 (1.300, 1.333) | 1.110 (1.094, 1.125) | 1.104 (1.089, 1.120) |
| Obesity | High SES score | 1 (reference) | 1 (reference) | 1 (reference) |
|  | Medium-high SES score | 1.270 (1.236, 1.305) | 1.251 (1.216, 1.286) | 1.230 (1.196, 1.265) |
|  | Medium SES score | 1.266 (1.199, 1.335) | 1.196 (1.131, 1.264) | 1.179 (1.115, 1.247) |
|  | Low SES score | 1.298 (1.195, 1.408) | 1.203 (1.105, 1.307) | 1.189 (1.092, 1.292) |
|  | Trend test | 1.153 (1.133, 1.173) | 1.131 (1.111, 1.151) | 1.121 (1.100, 1.141) |

Abbreviations: SES, socioeconomic status; OR, odds ratio; CI, confidence interval.

***Note*:** Model 1 was the unadjusted model; Model 2 adjusted for age and sex; Model 3 adjusted for age, sex, smoking habit, drinking habit, and physical activity level.

**Table S3. Correlation between gender-specific SES scores and prevalence of metabolic diseases**

| **Outcome** | **SES scores** | **Male** | | |  | **Female** | | |
| --- | --- | --- | --- | --- | --- | --- | --- | --- |
|  |  | **Model 1**  **OR (95% CI)** | **Model 2**  **OR (95% CI)** | **Model 3**  **OR (95% CI)** |  | **Model 1**  **OR (95% CI)** | **Model 2**  **OR (95% CI)** | **Model 3**  **OR (95% CI)** |
| Diabetes | SES score =3 | 1 (reference) | 1 (reference) | 1 (reference) |  | 1 (reference) | 1 (reference) | 1 (reference) |
|  | SES score =2 | 0.846 (0.815, 0.879) | 0.798 (0.767, 0.829) | 0.798 (0.767, 0.830) |  | 1.299 (1.263, 1.337) | 1.085 (1.054, 1.118) | 1.078 (1.047, 1.111) |
|  | SES score =1 | 0.907 (0.809, 1.014) | 0.738 (0.657, 0.828) | 0.738 (0.657, 0.828) |  | 1.704 (1.622, 1.789) | 0.966 (0.916, 1.018) | 0.964 (0.914, 1.015) |
|  | SES score =0 | 1.021 (0.864, 1.203) | 0.793 (0.669, 0.937) | 0.793 (0.669, 0.936) |  | 2.034 (1.892, 2.185) | 0.984 (0.912, 1.062) | 0.984 (0.912, 1.061) |
|  | Decrease per point | 0.904 (0.876, 0.932) | 0.839 (0.813, 0.866) | 0.840 (0.813, 0.867) |  | 1.287 (1.264, 1.310) | 1.005 (0.986, 1.025) | 1.004 (0.984, 1.023) |
|  | P for interaction | <0.001 | <0.001 | <0.001 |  |  |  |  |
| Dyslipidemia | SES score =3 | 1 (reference) | 1 (reference) | 1 (reference) |  | 1 (reference) | 1 (reference) | 1 (reference) |
|  | SES score =2 | 0.757 (0.731, 0.784) | 0.774 (0.747, 0.801) | 0.770 (0.744, 0.798) |  | 1.006 (0.982, 1.031) | 0.926 (0.904, 0.949) | 0.930 (0.908, 0.954) |
|  | SES score =1 | 0.680 (0.612, 0.755) | 0.732 (0.659, 0.813) | 0.729 (0.656, 0.810) |  | 1.116 (1.067, 1.167) | 0.859 (0.821, 0.900) | 0.863 (0.824, 0.904) |
|  | SES score =0 | 0.651 (0.555, 0.761) | 0.718 (0.612, 0.841) | 0.715 (0.609, 0.837) |  | 1.208 (1.129, 1.293) | 0.860 (0.801, 0.922) | 0.862 (0.803, 0.924) |
|  | Decrease per point | 0.794 (0.772, 0.817) | 0.816 (0.793, 0.840) | 0.814 (0.791, 0.838) |  | 1.048 (1.031, 1.065) | 0.936 (0.920, 0.952) | 0.938 (0.922, 0.954) |
|  | P for interaction | <0.001 | <0.001 | <0.001 |  |  |  |  |
| Hypertension | SES score =3 | 1 (reference) | 1 (reference) | 1 (reference) |  | 1(reference) | 1 (reference) | 1 (reference) |
|  | SES score =2 | 1.158 (1.119, 1.199) | 1.069 (1.032, 1.107) | 1.072 (1.034, 1.111) |  | 1.595 (1.557, 1.633) | 1.301 (1.268, 1.334) | 1.280 (1.248, 1.313) |
|  | SES score =1 | 1.296 (1.171, 1.435) | 0.984 (0.884, 1.095) | 0.989 (0.889, 1.101) |  | 2.254 (2.158, 2.354) | 1.139 (1.086, 1.194) | 1.133 (1.080, 1.188) |
|  | SES score =0 | 1.459 (1.251, 1.702) | 1.014 (0.865, 1.190) | 1.016 (0.866, 1.193) |  | 2.645 (2.474, 2.829) | 1.065 (0.991, 1.145) | 1.067 (0.992, 1.147) |
|  | Decrease per point | 1.148 (1.117, 1.181) | 1.037 (1.008, 1.068) | 1.039 (1.010, 1.070) |  | 1.473 (1.450, 1.497) | 1.101 (1.082, 1.121) | 1.096 (1.077, 1.115) |
|  | P for interaction | <0.001 | <0.001 | <0.001 |  |  |  |  |
| Obesity | SES score =3 | 1 (reference) | 1 (reference) | 1 (reference) |  | 1(reference) | 1 (reference) | 1 (reference) |
|  | SES score =2 | 0.971 (0.926, 1.018) | 0.995 (0.949, 1.044) | 0.990 (0.943, 1.039) |  | 1.464 (1.415, 1.515) | 1.391 (1.344, 1.441) | 1.363 (1.316, 1.411) |
|  | SES score =1 | 0.656 (0.556, 0.770) | 0.708 (0.600, 0.831) | 0.707 (0.598, 0.830) |  | 1.511 (1.424, 1.603) | 1.285 (1.208, 1.366) | 1.265 (1.190, 1.346) |
|  | SES score =0 | 0.882 (0.704, 1.093) | 0.979 (0.781, 1.215) | 0.974 (0.776, 1.208) |  | 1.508 (1.377, 1.648) | 1.220 (1.112, 1.337) | 1.209 (1.101, 1.325) |
|  | Decrease per point | 0.936 (0.900, 0.973) | 0.964 (0.927, 1.003) | 0.960 (0.923, 0.999) |  | 1.224 (1.199, 1.250) | 1.144 (1.119, 1.169) | 1.135 (1.110, 1.160) |
|  | P for interaction | <0.001 | <0.001 | <0.001 |  |  |  |  |

Abbreviations: SES, socioeconomic status; OR, odds ratio; CI, confidence interval.

***Note*:** Model 1 was the unadjusted model; Model 2 adjusted for age; Model 3 adjusted for age, smoking habit, drinking habit, and physical activity level.

**Table S4. Correlation between age-specific SES scores and risk of four metabolic diseases**

| **Outcome** | **SES scores** | **Age 40-59 years** | | |  | **Age ≥ 60 years** | | |
| --- | --- | --- | --- | --- | --- | --- | --- | --- |
|  |  | **Model 1**  **OR (95% CI)** | **Model 2**  **OR (95% CI)** | **Model 3**  **OR (95% CI)** |  | **Model 1**  **OR (95% CI)** | **Model 2**  **OR (95% CI)** | **Model 3**  **OR (95% CI)** |
| Diabetes | SES score =3 | 1 (reference) | 1 (reference) | 1 (reference) |  | 1 (reference) | 1 (reference) | 1 (reference) |
|  | SES score =2 | 1.111 (1.078, 1.145) | 1.044 (1.012, 1.077) | 1.035 (1.003, 1.068) |  | 0.831 (0.802, 0.861) | 0.824 (0.794, 0.854) | 0.823 (0.794, 0.854) |
|  | SES score =1 | 1.130 (1.044, 1.222) | 1.041 (0.961, 1.127) | 1.037 (0.957, 1.122) |  | 0.891 (0.842, 0.943) | 0.815 (0.768, 0.864) | 0.815 (0.769, 0.865) |
|  | SES score =0 | 1.277 (1.104, 1.472) | 1.074 (0.927, 1.241) | 1.072 (0.925, 1.238) |  | 0.963 (0.892, 1.039) | 0.869 (0.803, 0.940) | 0.871 (0.805, 0.942) |
|  | Decrease per point | 1.092 (1.066, 1.119) | 1.033 (1.008, 1.059) | 1.028 (1.002, 1.054) |  | 0.957 (0.937, 0.977) | 0.922 (0.902, 0.942) | 0.923 (0.903, 0.943) |
|  | P for interaction | <0.001 | <0.001 | <0.001 |  |  |  |  |
| Dyslipidemia | SES score =3 | 1 (reference) | 1 (reference) | 1 (reference) |  | 1 (reference) | 1 (reference) | 1 (reference) |
|  | SES score =2 | 0.917 (0.895, 0.939) | 0.914 (0.892, 0.937) | 0.915 (0.893, 0.938) |  | 0.789 (0.762, 0.816) | 0.765 (0.739, 0.792) | 0.769 (0.742, 0.796) |
|  | SES score =1 | 0.906 (0.849, 0.967) | 0.916 (0.858, 0.978) | 0.914 (0.856, 0.976) |  | 0.812 (0.769, 0.857) | 0.779 (0.736, 0.825) | 0.783 (0.740, 0.829) |
|  | SES score =0 | 0.992 (0.878, 1.120) | 0.951 (0.840, 1.074) | 0.945 (0.836, 1.068) |  | 0.827 (0.767, 0.891) | 0.798 (0.739, 0.861) | 0.801 (0.742, 0.865) |
|  | Decrease per point | 0.941 (0.922, 0.959) | 0.938 (0.920, 0.957) | 0.939 (0.920, 0.958) |  | 0.910 (0.892, 0.929) | 0.894 (0.875, 0.913) | 0.897 (0.878, 0.916) |
|  | P for interaction | 0.023 | 0.443 | 0.350 |  |  |  |  |
| Hypertension | SES score =3 | 1 (reference) | 1 (reference) | 1 (reference) |  | 1 (reference) | 1 (reference) | 1 (reference) |
|  | SES score =2 | 1.331 (1.298, 1.364) | 1.265 (1.233, 1.298) | 1.250 (1.218, 1.282) |  | 1.078 (1.041, 1.116) | 1.103 (1.065, 1.143) | 1.096 (1.058, 1.136) |
|  | SES score =1 | 1.161 (1.088, 1.239) | 1.067 (0.997, 1.140) | 1.062 (0.993, 1.135) |  | 1.255 (1.188, 1.327) | 1.108 (1.045, 1.174) | 1.104 (1.041, 1.170) |
|  | SES score =0 | 1.135 (1.001, 1.284) | 0.943 (0.830, 1.070) | 0.944 (0.831, 1.071) |  | 1.276 (1.182, 1.378) | 1.085 (1.003, 1.175) | 1.087 (1.004, 1.176) |
|  | Decrease per point | 1.196 (1.173, 1.220) | 1.136 (1.113, 1.160) | 1.128 (1.105, 1.151) |  | 1.098 (1.076, 1.121) | 1.044 (1.022, 1.067) | 1.043 (1.021, 1.066) |
|  | P for interaction | <0.001 | <0.001 | <0.001 |  |  |  |  |
| Obesity | SES score =3 | 1 (reference) | 1 (reference) | 1 (reference) |  | 1 (reference) | 1 (reference) | 1 (reference) |
|  | SES score =2 | 1.224 (1.183, 1.266) | 1.224 (1.183, 1.266) | 1.201 (1.160, 1.243) |  | 1.314 (1.252, 1.379) | 1.240 (1.181, 1.302) | 1.222 (1.163, 1.284) |
|  | SES score =1 | 1.092 (0.998, 1.193) | 1.100 (1.005, 1.203) | 1.090 (0.996, 1.192) |  | 1.327 (1.233, 1.427) | 1.212 (1.124, 1.308) | 1.197 (1.110, 1.292) |
|  | SES score =0 | 0.900 (0.747, 1.076) | 0.882 (0.731, 1.054) | 0.879 (0.729, 1.051) |  | 1.387 (1.257, 1.528) | 1.278 (1.155, 1.411) | 1.266 (1.144, 1.398) |
|  | Decrease per point | 1.119 (1.089, 1.149) | 1.118 (1.088, 1.148) | 1.105 (1.075, 1.135) |  | 1.134 (1.104, 1.164) | 1.096 (1.066, 1.126) | 1.090 (1.061, 1.121) |
|  | P for interaction | 0.483 | 0.459 | 0.382 |  |  |  |  |

Abbreviations: SES, socioeconomic status; OR, odds ratio; CI, confidence interval.

***Note*:** Model 1 was the unadjusted model; Model 2 adjusted for age and sex; Model 3 adjusted for age, sex, smoking habit, drinking habit, and physical activity level.

**Table S5. Associations of gender-specific marital status, living condition, and educational attainment with four metabolic diseases**

| **Outcome** |  | **Male** | | |  | **Female** | | |
| --- | --- | --- | --- | --- | --- | --- | --- | --- |
|  |  | **Model 1**  **OR (95% CI)** | **Model 2**  **OR (95% CI)** | **Model 3**  **OR (95% CI)** |  | **Model 1**  **OR (95% CI)** | **Model 2**  **OR (95% CI)** | **Model 3**  **OR (95% CI)** |
| Diabetes | **Educational attainment** |  |  |  |  |  |  |  |
|  | High school or above | 1 (reference) | 1 (reference) | 1 (reference) |  | 1 (reference) | 1 (reference) | 1 (reference) |
|  | Illiteracy, primary school, or middle school | 0.843 (0.812, 0.875) | 0.788 (0.759, 0.818) | 0.788 (0.758, 0.818) |  | 1.326 (1.291, 1.362) | 1.068 (1.038, 1.098) | 1.060 (1.031, 1.091) |
|  | **Living condition** |  |  |  |  |  |  |  |
|  | Living with a spouse or children | 1 (reference) | 1 (reference) | 1 (reference) |  | 1 (reference) | 1 (reference) | 1 (reference) |
|  | Living alone | 1.138 (1.020, 1.269) | 0.987 (0.883, 1.102) | 0.987 (0.882, 1.102) |  | 1.478 (1.403, 1.557) | 0.955 (0.904, 1.008) | 0.960 (0.909, 1.014) |
|  | **Marital status** |  |  |  |  |  |  |  |
|  | Married or having a partner | 1 (reference) | 1 (reference) | 1 (reference) |  | 1 (reference) | 1 (reference) | 1 (reference) |
|  | Single, divorced, separated or others | 1.056 (0.965, 1.154) | 0.911 (0.831, 0.997) | 0.911 (0.832, 0.998) |  | 1.390 (1.339, 1.442) | 0.919 (0.884, 0.956) | 0.923 (0.887, 0.961) |
| Dyslipidemia | **Educational attainment** |  |  |  |  |  |  |  |
|  | High school or above | 1 (reference) | 1 (reference) | 1 (reference) |  | 1 (reference) | 1 (reference) | 1 (reference) |
|  | Illiteracy, primary school, or middle school | 0.745 (0.720, 0.770) | 0.764 (0.738, 0.790) | 0.760 (0.734, 0.787) |  | 1.017 (0.994, 1.040) | 0.921 (0.900, 0.943) | 0.926 (0.904, 0.948) |
|  | **Living condition** |  |  |  |  |  |  |  |
|  | Living with a spouse or children | 1 (reference) | 1 (reference) | 1 (reference) |  | 1 (reference) | 1 (reference) | 1 (reference) |
|  | Living alone | 0.894 (0.807, 0.991) | 0.952 (0.858, 1.055) | 0.950 (0.857, 1.053) |  | 1.111 (1.058, 1.167) | 0.914 (0.869, 0.961) | 0.912 (0.868, 0.959) |
|  | **Marital status** |  |  |  |  |  |  |  |
|  | Married or having a partner | 1 (reference) | 1 (reference) | 1 (reference) |  | 1 (reference) | 1 (reference) | 1 (reference) |
|  | Single, divorced, separated or others | 0.852 (0.784, 0.926) | 0.904 (0.832, 0.983) | 0.903 (0.830, 0.981) |  | 1.132 (1.094, 1.171) | 0.945 (0.913, 0.979) | 0.944 (0.912, 0.978) |
| Hypertension | **Educational attainment** |  |  |  |  |  |  |  |
|  | High school or above | 1 (reference) | 1 (reference) | 1 (reference) |  | 1 (reference) | 1 (reference) | 1 (reference) |
|  | Illiteracy, primary school, or middle school | 1.171 (1.133, 1.211) | 1.068 (1.031, 1.106) | 1.071 (1.034, 1.109) |  | 1.680 (1.643, 1.719) | 1.317 (1.285, 1.349) | 1.295 (1.264, 1.327) |
|  | **Living condition** |  |  |  |  |  |  |  |
|  | Living with a spouse or children | 1 (reference) | 1 (reference) | 1 (reference) |  | 1 (reference) | 1 (reference) | 1 (reference) |
|  | Living alone | 1.183 (1.070, 1.308) | 0.958 (0.862, 1.064) | 0.959 (0.863, 1.066) |  | 1.501 (1.432, 1.574) | 0.852 (0.809, 0.897) | 0.865 (0.821, 0.911) |
|  | **Marital status** |  |  |  |  |  |  |  |
|  | Married or having a partner | 1 (reference) | 1 (reference) | 1 (reference) |  | 1 (reference) | 1 (reference) | 1 (reference) |
|  | Single, divorced, separated or others | 1.167 (1.077, 1.266) | 0.951 (0.873, 1.036) | 0.956 (0.878, 1.041) |  | 1.474 (1.426, 1.523) | 0.878 (0.846, 0.910) | 0.888 (0.856, 0.921) |
| Obesity | **Educational attainment** |  |  |  |  |  |  |  |
|  | High school or above | 1 (reference) | 1 (reference) | 1 (reference) |  | 1 (reference) | 1 (reference) | 1 (reference) |
|  | Illiteracy, primary school, or middle school | 0.962 (0.918, 1.007) | 0.990 (0.944, 1.037) | 0.984 (0.938, 1.032) |  | 1.514 (1.466, 1.564) | 1.429 (1.382, 1.477) | 1.398 (1.353, 1.445) |
|  | **Living condition** |  |  |  |  |  |  |  |
|  | Living with a spouse or children | 1 (reference) | 1 (reference) | 1 (reference) |  | 1 (reference) | 1 (reference) | 1 (reference) |
|  | Living alone | 0.837 (0.719, 0.969) | 0.888 (0.763, 1.028) | 0.889 (0.763, 1.029) |  | 1.017 (0.951, 1.086) | 0.877 (0.819, 0.937) | 0.886 (0.827, 0.947) |
|  | **Marital status** |  |  |  |  |  |  |  |
|  | Married or having a partner | 1 (reference) | 1 (reference) | 1 (reference) |  | 1 (reference) | 1 (reference) | 1 (reference) |
|  | Single, divorced, separated or others | 0.781 (0.689, 0.881) | 0.825 (0.728, 0.932) | 0.828 (0.730, 0.935) |  | 1.051 (1.004, 1.100) | 0.915 (0.873, 0.959) | 0.921 (0.879, 0.966) |
|  |  |  |  |  |  |  |  |  |

Abbreviations: OR, odds ratio; CI, confidence interval.

***Note*:** Model 1 was the unadjusted model; Model 2 adjusted for age; Model 3 adjusted for age, smoking habit, drinking habit, and physical activity level.
